# Second-Line Therapies in Primary Biliary Cholangitis: A Comparative Review of Obeticholic Acid, Fibrates, Seladelpar, and Elafibranor

**DOI:** 10.3390/biomedicines13102335

**Published:** 2025-09-24

**Authors:** Fares Jamal, Amani Elshaer, Mayar H. Alatout, Nour B. Odeh, Amal Youssef, Humam Abo Abdullah, Sandra Elmasry, Tala Shahin, Hussein Abdul Nabi, Astin R. Worden, Talha A. Malik, Blanca C. Lizaola-Mayo

**Affiliations:** 1 Department of Hematology and Oncology, Mayo Clinic, 5777 E. Mayo Blvd., Phoenix, AZ 85054, USA; 2 Department of Gastroenterology and Hepatology, Mayo Clinic, Phoenix, AZ 85054, USA; 3 Department of Surgery, Mayo Clinic, Phoenix, AZ 85054, USA; 4 Department of Cardiology, Mayo Clinic, Phoenix, AZ 85054, USA; youssef.amal@mayo.edu (A.Y.);; 5 Department of Medicine, Mayo Clinic, Phoenix, AZ 85054, USA

**Keywords:** primary biliary cholangitis, second-line therapy, obeticholic acid, fenofibrate, seladelpar, elafibranor

## Abstract

Primary biliary cholangitis (PBC) is a chronic autoimmune liver disease marked by cholestasis and progressive fibrosis. While ursodeoxycholic acid (UDCA) remains the first-line therapy, approximately 30–40% of patients have an inadequate biochemical response, increasing the risk of disease progression. Obeticholic acid (OCA), a potent farnesoid X receptor (FXR) agonist, was the first second-line agent approved by the only Food and Drug Administration (FDA) and has demonstrated moderate biochemical efficacy but limited tolerability due to dose-dependent pruritus and safety concerns in cirrhosis. Fenofibrate, a peroxisome proliferator-activated receptor alpha (PPAR-α) agonist, showed substantial alkaline phosphatase (ALP) reductions when added to UDCA, although its long-term benefit remains unconfirmed in large-scale trials and its use remains off-label in the United States, unlike FDA-approved agents. Seladelpar, a selective peroxisome proliferator-activated receptor delta (PPAR-δ) agonist, and elafibranor, a dual PPAR-α/δ agonist, have both recently received FDA accelerated approval after demonstrating significant improvements in ALP, biochemical response rates, and pruritus relief in phase 3 trials. This review summarizes these second-line therapies’ mechanisms, efficacy, safety, and limitations emphasizing the need for individualized treatment decisions and ongoing research into long-term clinical outcomes.

## 1. Introduction

Primary Biliary Cholangitis (PBC) is a common autoimmune liver disease, with a global prevalence ranging from 1.9 to 40.2 cases per 100,000 and incidence estimates between 0.3 and 5.8 per 100,000 per year, depending on geography [1,2]. It primarily affects middle-aged women, with a prevalence of at least one in 1000 women over the age of 40 [3,4]. PBC is characterized by progressive destruction of the interlobular bile ducts, leading to cholestasis, fibrosis, and eventually biliary cirrhosis [5]. The diagnosis is typically suspected in patients with elevated liver enzymes, particularly alkaline phosphatase (ALP) and gamma-glutamyl transferase (GGT), along with the presence of circulating antimitochondrial antibodies (AMA) [5]. Despite advances in therapy, PBC remains a progressive disease that can lead to cirrhosis, liver failure, and need for transplantation, representing a significant unmet need in hepatology [6].

According to the American Association for the Study of Liver Diseases (AASLD), ursodeoxycholic acid (UDCA) is the first-line treatment for PBC [7]. Several trials have shown that UDCA prolongs transplant-free survival and slows disease progression; however, it does not reliably control pruritus and fatigue [8,9]. Furthermore, approximately 30–40% of patients treated with UDCA continue to have elevated ALP and bilirubin levels after 12 months, which is associated with an increased risk of disease progression [10]. Additionally, patients with advanced fibrosis or cirrhosis may have limited response to PBC treatment with UDCA [11]. Therefore, the AASLD guidelines recommend treatment reassessment in 12 months following starting UDCA.

**Figure 1:**
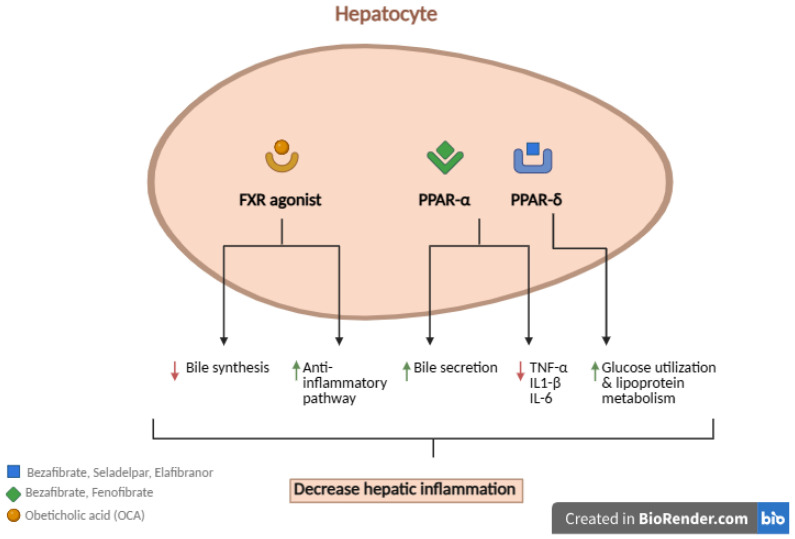
Mechanisms of Action of Novel Agents in Primary Biliary Cholangitis.

If there is an inadequate biochemical response, second-line treatments should be considered [12]. In May 2016, obeticholic acid (OCA) was approved as a second-line agent by the Food and Drug Administration (FDA). However, clinical studies have shown promise in other agents, including fibrates (bezafibrate, fenofibrate), seladelpar, and elafibranor (Table 1) [13]. These treatments act through distinct mechanisms of action (Figure 1). This review provides an overview of second-line treatment options for PBC, with emphasis on their clinical practice implications, efficacy and safety.

## 2. Obeticholic Acid

### 2.1. Mechanism of Action

OCA is a semi-synthetic hydrophobic bile acid analog derived from chenodeoxycholic acid that functions as a highly selective agonist of the farnesoid X receptor (FXR) [25]. FXR, a nuclear bile acid receptor, plays a central role in regulating bile acid homeostasis and stimulates the secretion of gut-derived hormones, particularly fibroblast growth factor 19 [26,27]. Additionally, FXR signaling protects hepatocytes from bile acid toxicity by inhibiting bile acid synthesis and promoting choleresis through upregulation of bile acid transporters. It also activates anti-inflammatory and antifibrotic pathways leading to reduced hepatic inflammation; thus, making it a promising therapeutic agent for PBC [27].

### 2.2. Clinical Trials & Efficacy

Several clinical trials have investigated the impact of OCA on PBC. In a multicenter observational single-arm study, D’Amato et al. (2021) evaluated 191 patients from 38 secondary and tertiary care centers, with follow-up extending to at least 12 months [28]. At 12 months, significant median reductions were observed in ALP (−32.3%), alanine aminotransferase (ALT) (−31.4%), and bilirubin (−11.2%). Patients with cirrhosis showed a lower response rate than those without cirrhosis (29.5% vs. 49.2%; *p* = 0.01), largely due to a higher discontinuation rate in this group (30% vs. 12%; *p* = 0.004), although both groups exhibited similar reductions in ALP levels (29.4% vs. 34%; *p* = 0.53). Patients with PBC- autoimmune hepatitis (AIH) overlap had comparable response rates to those with PBC alone (46.4% vs. 42.3%; *p* = 0.68) but experienced greater reductions in ALT at 6 months (−38% vs. −29%; *p* = 0.04). Overall, OCA was associated with reductions in ALP, ALT, and bilirubin at 12 months across the study population.

These findings align with those of the POISE trial, a randomized, double-blind, placebo-controlled phase 3 study that evaluated OCA over a 12-month period [27]. In this study, Nevens et al. enrolled 217 patients with PBC who had either an inadequate response to UDCA or were unable to tolerate its side effects. These patients were administered OCA as part of the study protocol [27]. Participants were randomly assigned to receive either 10 mg of OCA daily (n = 73), 5 mg of OCA with the option to up-titrate to 10 mg (n = 70), or placebo (n = 73). The primary endpoint was an ALP level of less than 1.67 times the upper limit of normal, with a reduction of at least 15% from baseline, and a normal total bilirubin level. Among those randomized and treated, 93% continued UDCA as background therapy. The primary endpoint was achieved by a higher proportion of patients in the 5–10 mg group (46%) and the 10 mg group (47%) compared to the placebo group (10%) (*p* < 0.001 for both comparisons). Additionally, the proportion of patients achieving at least a 15% reduction in ALP from baseline was significantly higher in both the 5–10 mg group (77%) and the 10 mg group (77%) than in the placebo group (29%) (*p* < 0.001 for both comparisons). Total bilirubin levels decreased in both OCA treatment groups, while a progressive increase was observed in the placebo group (*p* < 0.001 for both comparisons). At 12 months, ALP levels were reduced by a mean of 113 ± 14 U/L in the 5–10 mg OCA group and 130 ± 15 U/L in the 10 mg group, compared to 14 ± 15 U/L in the placebo group (*p* < 0.001 for both comparisons). Patients receiving OCA continued to have reductions in ALP and total bilirubin over a two-year period, indicating a sustained treatment effect. However, no significant differences were observed between either OCA group and placebo in non-invasive assessments of liver fibrosis at 12 months. Changes in transient elastography and enhanced liver fibrosis (ELF) scores from baseline to 12 months were not significantly different between treatment groups (*p* = 0.6818 and *p* = 0.4718 for 5–10 mg OCA vs. placebo; *p* = 0.3267 and *p* = 0.2324 for 10 mg OCA vs. placebo, respectively [27].

In a single-arm, prospective multicenter study, Gomez et al. (2020) evaluated the effectiveness and safety of OCA in 120 adult patients with PBC across 18 different centers [14]. The study included participants who had either an inadequate response or intolerance to UDCA, or showed disease progression to advanced fibrosis despite an adequate biochemical response. Most patients (65%) were followed for at least 12 months after initiating OCA therapy. Based on the POISE criteria, 29.5% of patients achieved a treatment response. Significant reductions in ALP, ALT, and bilirubin were observed across the cohort during the study period, with a mean decrease in ALP of 81.3 U/L (95% CI: 42.5–120.0; *p* < 0.001), ALT decrease of 22.1 U/L (95% CI: 10.4–33.8; *p* < 0.001), and bilirubin decrease of 0.12 mg/dL (95% CI: 0–0.24; *p* = 0.044). However, surrogate markers of liver fibrosis, such as the fibrosis-4 (FIB-4) index and aspartate aminotransferase (AST) to Platelet Ratio Index (APRI), remained unchanged over the 12-month treatment period. Transient elastography findings indicated that liver fibrosis remained stable during OCA therapy [14].

Based on the POISE trial results, OCA received approval as a second-line treatment for PBC in patients with an inadequate response or intolerance to UDCA [29,30]. Subsequent extension studies have confirmed the durability of its biochemical benefits and its suitability as monotherapy in UDCA-intolerant patients [31].

### 2.3. Safety Profile & Limitations

The use of OCA presents several limitations, including its high cost, restricted use in patients with cirrhosis, and notable side effects. Pruritus, a frequent adverse effect, occurred in a dose-dependent manner, though its underlying mechanism remains unclear [27]. In a study by John et al. (2021), OCA use in patients with PBC-related cirrhosis was associated with a 3.9-fold increase in the risk of hepatic decompensation [32]. This effect is likely related to impaired hepatic clearance in cirrhosis, leading to drug accumulation and excessive FXR activation, which can worsen portal hypertension and cholestatic injury in vulnerable livers [32]. However, larger prospective trials are needed to validate these findings, and such studies are currently underway.

These safety concerns led the FDA to revise the drug’s labeling in 2021, advising against its use in patients with cirrhosis who have current or past signs of decompensation, including hepatic encephalopathy, ascites, or variceal bleeding, or in those with evidence of portal hypertension, such as gastroesophageal varices or persistent thrombocytopenia [31]. AASLD further emphasized that OCA is contraindicated in patients with decompensated cirrhosis and recommended close monitoring of all patients with cirrhosis receiving OCA, even those without advanced disease [18]. According to these guidelines, OCA is contraindicated in individuals with Child-Pugh Class B or C cirrhosis, as well as in Child-Pugh A patients exhibiting any signs of portal hypertension. In patients with compensated cirrhosis without portal hypertension, OCA may be used cautiously, but the dose should not exceed 5 mg per day [12]. Given these concerns, careful patient selection and close monitoring are essential when initiating OCA in individuals with cirrhosis. In such cases, managing pruritus before and during treatment may help improve tolerability and treatment outcomes [28]. Furthermore, at its current annual cost of approximately $69,000, the therapy was not cost-effective, with an incremental cost-effectiveness ratio of $473,400 per quality-adjusted life year (QALY). Pricing reductions to below $18,450 per year would be necessary to meet standard willingness-to-pay thresholds [29].

## 3. Peroxisome Proliferator-Activated Receptor-Alpha Agonists

### 3.1. Mechanism of Action

Fibrates including, fenofibrate, gemfibrozil, and bezafibrate, act through activating peroxisome proliferator-activated receptor alpha (PPAR-α), a nuclear receptor involved in lipid metabolism and inflammation [33]. PPAR-α activation enhances bile acid metabolism and secretion [33,34]. PRAR-α decreases inflammation through downregulating pro-inflammatory cytokines and chemokines such as tumor necrosis factor alpha (TNF-α), IL-1β, IL-6, and other cytokines, partly through inhibiting nuclear factor kappa-light-chain-enhancer of activated B cells (NF-κB) signaling. These effects were shown in both clinical and experimental settings [35,36,37].

The fibrates modulate hepatic stellate cell activation; as it regulates bile acid synthesis and detoxification, and phospholipid secretion [7,38]. These effects collectively reduce cholesterol and hepatic inflammation, and it reflects by reductions in serum ALP and GGT levels [7,15,39].

### 3.2. Clinical Trials & Efficacy

The strongest evidence for fibrates in PBC comes from the BEZURSO trial, a 24-month, double-blind, placebo-controlled phase 3 study that randomized 100 patients with UDCA-incomplete response to bezafibrate (400 mg/day, n = 50) or placebo (n = 50), in addition to UDCA. The primary endpoint, biochemical response at 24 months, was achieved in 31% of the bezafibrate group versus 0% of the placebo group (*p* < 0.001). ALP normalization occurred in 67% compared with 2% in placebo (*p* < 0.001). Improvements were also noted in pruritus, fatigue, and non-invasive fibrosis markers (liver stiffness and ELF score). However, differences in clinical outcomes such as liver decompensation were not statistically significant. Adverse events included higher rates of myalgia (20% vs. 10%) and a modest increase in serum creatinine [40]. This pivotal phase 3 trial established bezafibrate as a highly effective adjunct to UDCA in PBC, though it remains off-label in the United States.

There is growing evidence that adding fenofibrate to UDCA in patients with UDCA-resistant PBC significantly improves biochemical markers, especially ALP. A recent randomized clinical trial on 117 treatment-naive PBC patients compared UDCA and fenofibrate (200 mg daily) with UDCA monotherapy [15]. Among the participants, 60 received UDCA alone and 57 received the combination therapy. The primary outcome, biochemical response at 12 months according to the Barcelona criterion, was achieved in 46/57 patients (81.4%) in the UDCA–fenofibrate group and 36/60 patients (64.3%) in the UDCA-only group, with a statistically significant difference (*p* = 0.048) [15]. However, there were no statistical differences between the UDCA–fenofibrate and UDCA-only groups in liver stiffness measured by transient elastography (*p* = 0.335), FIB-4 score (*p* = 0.692), and GGT (*p* = 0.605) [15].

An open-label study included 20 patients with ALP levels greater than twice the upper limit of normal (ULN), which was defined as 130 U/L, after UDCA treatment [37]. These patients received fenofibrate 160 mg/day for 48 weeks [37]. Median ALP levels dropped from 351 U/L (range 214–779) at baseline to 177 U/L (range 60–384) after 48 weeks of treatment (*p* < 0.0001) [37].

Fenofibrate has shown efficacy in reducing pruritus in PBC patients, although the evidence is less robust than for bezafibrate [22]. The AASLD notes that fibrates, including fenofibrate, may improve pruritus in PBC, based on observational studies and clinical experience, but most high-quality randomized controlled trial data specifically address bezafibrate rather than fenofibrate [7].

Importantly, while fenofibrate is more commonly used in the United States, most high-quality randomized data come from bezafibrate studies in Europe and Japan. The difference may relate to pharmacologic profiles: fenofibrate is a selective PPAR-α agonist, whereas bezafibrate acts as a pan-PPAR agonist (α, δ, γ), which may account for broader biochemical and symptomatic benefits observed with bezafibrate [41]. Currently, fibrates are only approved by the FDA as lipid-lowering medications in the United States (US) and multiple countries rather than PBC treatment [7]. The AASLD considers fibrates an off-label option for PBC patients with incomplete UDCA response but advises against their use in decompensated liver disease (Child-Pugh B and C) due to lack of safety data and potential risk of hepatotoxicity [7].

### 3.3. Safety Profile and Limitations

Fenofibrate may cause reversible elevations in serum creatinine, likely due to increased creatinine production rather than a true decline in the glomerular filtration rate [7,42]. In a randomized trial, creatinine and transaminases levels in the UDCA-fenofibrate dual therapy group increased within the first month, then returned to normal, and remained stable thereafter until the end of the study, even in patients with cirrhosis [15]. In the Fenofibrate Intervention and Event Lowering in Diabetes (FIELD) trial, the average creatinine elevation was 12% which was reversible once fenofibrate was discontinued [43]. Therefore, in patients with mild to moderate renal impairment, dose adjustment is required, but use is absolutely contraindicated in severe renal dysfunction (estimated glomerular filtration rate (eGFR) < 30 mL/min/1.73 m^2^). Additionally, the risk of myopathy may increase with the use of fibrates and renal dysfunction or concomitant statin use [42].

The AASLD notes that fenofibrate can cause transient elevations in transaminases, but not associated with any toxicity and normalize with therapy discontinuation [7]. An increase in bilirubin can occur. This has been attributed to competitive inhibition of the transporter organic-anion-transporting polypeptide, which transports both bile acids and bilirubin, among other endogenous substances [7]. Although fibrates, particularly bezafibrate and fenofibrate, have demonstrated biochemical improvements in patients with PBC, several limitations restrict their routine use. These agents are not FDA-approved for PBC, and most clinical evidence is derived from small, non-U.S. studies with limited follow-up. Long-term data on outcomes such as progression to cirrhosis, need for liver transplantation, and overall survival remain sparse.

## 4. Seladelpar

### 4.1. Mechanism of Action

Seladelpar is a selective peroxisome proliferator-activated receptor delta (PPAR-δ) agonist. A nuclear receptor that is expressed in hepatic stellate cells, hepatocytes, cholangiocytes, and kupffer cells, all of which are involved in PBC pathogenesis [44]. PPAR-δ activation by seladelpar results in bile acid synthesis suppression, through downregulating cholesterol 7α-hydroxylase (CYP7A1), which is mediated by fibroblast growth factor 21 (FGF21) signaling [45]. Consequently, resulting in bile acid homeostasis improvement, hepatic inflammation, and fibrosis reduction. Additionally, it has anti-inflammatory effects on hepatic macrophages and stellate cells, which in turn reduces pro-inflammatory cytokines and fibrogenic activity [44,45,46].

### 4.2. Clinical Trials & Efficacy

A double-blind, randomized, placebo-controlled phase 3 study, the ENHANCE trial, investigated patients with PBC who responded inadequately or did not tolerate UDCA. Patients who were on UDCA for PBC were randomized 1:1:1 to seladelpar 5 mg (n = 89), seladelpar 10 mg (n = 89), or placebo (n = 87). The primary as well as the secondary endpoints were then analyzed at 3 months because of early termination. The primary endpoint at month 3 was the composite biochemical response defined as: ALP < 1.67 times the ULN, ≥15% decrease in ALP from baseline levels, and total bilirubin ≤ ULN. Among the patients analyzed at 3 months, 78.2% of those who received seladelpar 10 mg (n = 55) and 57.1% of those who received seladelpar 5 mg (n = 56) achieved a composite biochemical response, compared to only 12.5% in the placebo group (n = 56) (*p* < 0.0001). The proportion of patients who achieved ALP < 1.67 × ULN at 3 months was higher in the 10 mg seladelpar group (95%) and the 5 mg seladelpar group (95%), compared to placebo (23%) (*p* < 0.0001 in both comparisons). As for the secondary endpoints of the study, they included both decrease in ALT from baseline and mean pruritic numerical rating scale (NRS) change baseline NRS ≥ 4 (placebo n = 18, seladelpar 5 mg n = 17, and seladelpar 10 mg, n = 18). The mean pruritis NRS change was −2.01 in seladelpar 5 mg (*p* = 0.48) and −3.14 in 10 mg (*p* = 0.02) when compared to −1.55 placebo. ALT decrease from baseline was reported as −16.7% in seladelpar 10 mg (*p* < 0.03) and −23.4% in seladelpar 5 mg (*p* = 0.0008) when compared to placebo of −4.0% [16].

Additionally, the RESPONSE trial, a 12-month placebo-controlled double-blinded phase 3 trial also investigated the efficacy and safety of seladelpar. Randomization was performed in a 2:1 ratio to 10 mg of seladelpar (n = 128) or placebo (n = 65), where 93.8% of patients were still on background UDCA. The primary endpoint at 12 months was a composite biochemical response, defined identically to the ENHANCE trial. Key secondary endpoints included normalization of ALP at 12 months and mean change in pruritis NRS score measured at 6 months among patients with baseline NRS ≥ 4. An additional secondary endpoint measured was the ALT normalization at 12 months. 79/128 patients (61.7%) who received 10 mg seladelpar achieved a composite response, compared to 13/65 patients (20.0%) in the placebo group (*p* < 0.001). 32 out of 128 patients (25%) of the seladelpar group had normalization in ALP levels compared to 0% in the placebo group (*p* < 0.001). Those on seladelpar 10 mg (n = 49) achieved −3.2 points while patients on placebo (n = 23) achieved −1.7 points (*p* = 0.005). Furthermore, 56.3% achieved normalization in ALT level at 12 months in the 10 mg seladelpar group compared to 25% in the placebo group (*p* < 0.01). There were no significantly observed changes in liver stiffness or liver fibrosis at 12 months. However, ALT and GGT were significantly decreased with seladelpar which indicates reduced hepatocellular injury [47].

The ASSURE trial is an ongoing open-label extension study that is evaluating the long-term safety and efficacy of seladelpar 10 mg in patients who had previously participated in clinical trials including ENHANCE and RESPONSE. Patients were enrolled into two main sub-groups; those who received seladelpar previously and those who had received placebo or alternative treatment (initiated seladelpar de novo). The endpoints were evaluated at 12 and 24 months. The primary endpoints were composite biochemical response, ALP normalization, and pruritic improvement. Among patients from the RESPONSE trial who continued seladelpar, at 12 months 38/53 patients (72%) sustained a composite biochemical response. As for the group that had originally received placebo in RESPONSE trial but was then started on seladelpar de novo in ASSURE trial, 51/54 patients (94%) newly achieved the composite endpoint at 12 months. For ALP normalization, in the seladelpar continuation group, 9/53 (17%) achieved normalization at month 12 and in the crossover cohort, 27/54 (50%) achieved normalization at month 12. At 24 months, across the broader population that included both RESPONSE trial and legacy phase 2 patients (n ≈ 145), composite biochemical response rates were sustained in 70–73%, while ALP normalization increased further to 42% (n ≈ 61/145). These data illustrate continued biochemical improvement with prolonged seladelpar exposure. Regarding symptoms, in patients who had previously received seladelpar (n = 27), the mean decrease in pruritus NRS at month 18 was −3.5 to −3.8 points. New starters on seladelpar in ASSURE trial showed similar pruritus improvements after initiation. As for liver injury markers, ALT, AST, and GGT levels remained stable or continued to decline throughout the 24 months. The rate of ALT normalization in patients who had elevated baseline ALT was not explicitly reported in ASSURE trial but was described as sustained or improved over time [23].

Seladelpar achieved the Breakthrough Therapy Designation from the FDA for PBC considering the improvement over current approved drugs. Approval was accelerated in the US in 2024 for adults with PBC that experienced intolerance or incomplete response to UDCA [48]. A new Drug Application (NDA) was approved in the US and regulatory review is ongoing in Europe and the United Kingdom as of 2024 [24].

### 4.3. Safety Profile and Limitations

Overall, seladelpar was well tolerated. Adverse events were almost identical to placebo when observed in both short and long-term studies [16,23,47]. The side effects that were more commonly reported in the study group were nausea, gastrointestinal discomfort, and mild headaches [16,23,47]. As detailed earlier, pruritus was less frequent in seladelpar when compared to placebo. There were no reported treatment-related deaths [16,23,47]. Pricing information for seladelpar is not widely available in the literature. As with many orphan-designated or rare disease therapies, initial access challenges are anticipated, including high out-of-pocket costs, variable insurance coverage, and the need for payer prior authorizations, which may delay initiation in clinical practice. Considering it is a drug that has been newly approved for an uncommon disease, it is expected to be similarly priced to second-line PBC agents; cost-effectiveness analyses and reimbursement details are pending broader market release [24]. Although seladelpar showed improvement in biochemical markers and symptom relief for PBC, there are still unfilled gaps in understanding its long-term clinical impact. There is limited data on outcomes like progression to cirrhosis, eventual need for liver transplantation, and overall survival rates. This data is still being studied, and there is no clear evidence available that this drug can reverse fibrosis that is already established. Most of the detailed clinical trials above excluded patients suffering from decompensated cirrhosis or advanced liver failure. This limits information regarding seladelpar’s safety and efficacy in higher-risk groups. Additionally, since it is a newly approved therapy, patients might face insurance coverage challenges [45,46].

Contraindications for this drug include decompensated cirrhosis, such as those experiencing ascites, hepatic encephalopathy or active variceal bleeding [20]. It should also be avoided in patients with biliary obstruction. When using concurrent cytochrome P450 2C9 (CYP2C9) inhibitors, organic anion transporters 3 (OAT3) inhibitors, or bile acid sequestrants, seladelpar should be used with extra caution as those drugs can ultimately alter the body’s exposure to the drug [20]. In patients with cirrhosis, close monitoring is recommended throughout treatment [46].

## 5. Elafibranor

### 5.1. Mechanism of Action

Elafibranor functions as an agonist of PPAR-α/δ [49]. It has been shown to enhance insulin sensitivity, improve glucose homeostasis, modulate lipid metabolism, and exert anti-inflammatory effects [49]. Mechanistic pathways activated by PPAR-α and PPAR-δ have been described above.

### 5.2. Clinical Trials & Efficacy

A phase 2 trial evaluated the long-term safety of elafibranor and its effects on clinical outcomes [17]. In this 12-week, double-blind phase II trial, 45 adults with PBC and an incomplete response to UCDA were enrolled. Participants were randomly assigned to receive elafibranor 80 mg (n = 15), elafibranor 120 mg (n = 15), or placebo (n = 15). The primary endpoint was the relative change in ALP, aiming for a level below 1.67 × ULN at week 12. At 12 weeks, ALP was reduced by −48.3 ± 14.8% in the elafibranor 80 mg and by −40.6 ± 17.4% in the elafibranor 120 mg group compared to a +3.2 ± 14.8% increase in the placebo group (*p* < 0.001 for both comparisons) [17]. The composite endpoint, comprising ALP ≤ 1.67 times the ULN, a reduction in ALP of >15%, and total bilirubin within the normal range, was achieved in 67% of patients in the elafibranor 80 mg group and 79% in the 120 mg group, compared to 6.7% in the placebo group [17]. Furthermore, elafibranor treatment was associated with marked decrease in inflammatory and immunologic biomarkers, including high-sensitivity C-reactive protein (hsCRP), immunoglobulin M (IgM), and circulating levels of the bile acid precursor 7α-hydroxy-4-cholesten-3-one (C4) [17]. elafibranor did not induce or exacerbate pruritus, and patients who experienced pruritus at baseline reported a reduction in pruritic symptoms by the end of treatment [17]. GGT, hsCRP, and IgM were significantly reduced during treatment [17]. These markers returned toward baseline levels after treatment discontinuation [17]. This study represents the first clinical evidence demonstrating the therapeutic efficacy of elafibranor, a dual PPARα/PPARδ agonist, in patients with PBC who exhibit an incomplete response to UDCA [17].

ELATIVE, a phase 3 trial, enrolled 161 patients [50]. The primary endpoint was a biochemical response at week 52, indicated by an ALP level less than 1.67 times the ULN, accompanied by a reduction of ≥15% from baseline and a total bilirubin level at or below the ULN. Key secondary endpoints included normalization of ALP levels at week 52 and changes in pruritus intensity from baseline through week 52 and week 24, assessed using the worst itch numerical rating scale (WI-NRS) among patients with moderate-to-severe pruritus (defined as a baseline WI-NRS score ≥ 4) [50]. Additional secondary endpoints included changes from baseline to week 52 in patient-reported outcomes related to pruritus, evaluated using the Primary Biliary Cholangitis-40 (PBC-40) questionnaire and the 5-D itch scale.

A biochemical response, the primary endpoint, was observed in 51% of patients (55 out of 108) treated with elafibranor, compared to 4% (2 out of 53) in the placebo group, representing a 47-percentage point difference (*p* < 0.001) [50]. Normalization of ALP levels at week 52 occurred in 15% of patients receiving elafibranor and in none of the patients receiving placebo (*p* < 0.002) [50]. Among patients with moderate-to-severe pruritus (44 in the elafibranor group and 22 in the placebo group), the least-squares mean change in WI-NRS score from baseline through week 52 did not differ significantly between the two groups. Side effects more frequently reported in the elafibranor group included abdominal pain, diarrhea, nausea, and vomiting [50]. However, fibrosis-related endpoints such as transient elastography and non-invasive fibrosis scores were neutral in this trial. This highlights the need for longer-term studies to confirm the impact of elafibranor on cirrhosis, liver transplantation, and survival.

Elafibranor received accelerated approval in 2024 as a second-line therapy for adults with PBC who have an inadequate response or intolerance to UDCA, based on results from the phase 3 ELATIVE trial demonstrating significant biochemical response and favorable tolerability, including no worsening of pruritus [50,51]. However, it is not yet formally incorporated into AASLD or EASL guidelines.

### 5.3. Safety & Limitations

Elafibranor was generally well tolerated, and no deaths were reported during the study [17]. No serious adverse events were observed in the placebo group or among patients receiving elafibranor 80 mg [17]. In contrast, two patients in the 120 mg group experienced at least one adverse event of severe intensity, with one patient developing an ischemic stroke within 24 h of the first dose [17]. In the phase II trial, elevations in aminotransferases were observed, including one case in the 120 mg group possibly related to an AIH flare [17]. Rhabdomyolysis and acute kidney injury occurred in a patient with cirrhosis on elafibranor and statin therapy [17]. Myalgia or creatine phosphokinase (CPK) elevation was reported, particularly when combined with statins [17]. Supporting the renal safety of elafibranor in the phase 2b trial, circulating levels of cystatin C, a more accurate marker of glomerular filtration, remained unaffected by elafibranor treatment [17].

Elafibranor should be avoided in patients with complete biliary obstruction. If biliary obstruction is suspected, treatment should be interrupted and appropriate clinical management initiated [21]. Based on findings from animal reproduction studies, elafibranor may cause fetal harm when administered during pregnancy [21]. Hence, elafibranor should not be prescribed for pregnant patients.

The majority of patients enrolled in Elative trial were white [50]. Although this reflects the known epidemiology of PBC, racial minorities were underrepresented, and ethnicity data were not recorded [50]. Primary outcomes in current trials have relied on biochemical markers such as ALP and bilirubin rather than direct clinical endpoints. While these are regulatory-accepted surrogate markers, they do not fully reflect disease progression or quality of life. Patients with advanced liver disease (e.g., decompensated cirrhosis) or autoimmune overlap syndromes were excluded from the study. This limits the generalizability of findings to the broader and more clinically diverse PBC population.

## 6. Conclusions

Despite important progress, the evidence base for second-line therapies in PBC is still largely driven by surrogate biochemical endpoints, with limited long-term data on fibrosis progression, transplantation, or survival. This limitation constrains firm treatment algorithms and underscores the need for ongoing comparative and outcome-based research. From a comparative perspective, OCA and fibrates both achieve ALP reduction, but fibrates often provide greater biochemical response and potential pruritus relief, whereas OCA is limited by dose-dependent pruritus and contraindications in cirrhosis [15,22,27]. On the other hand, seladelpar (PPAR-δ selective) consistently improves pruritus and shows high biochemical response rates, while elafibranor (dual PPAR-α/δ) improves biochemical endpoints with a neutral pruritus effect but a reassuring safety signal overall [16,50]. However, because elafibranor does not improve pruritus, this may limit its adoption in clinical practice compared with seladelpar, particularly for patients in whom symptom control is a major therapeutic goal. These distinctions highlight that drug choice must be individualized based on patient priority, symptom burden, comorbidities, and safety considerations, and treatment sequencing will likely evolve rapidly as additional real-world data become available.

In summary, second-line therapies for PBC have evolved significantly, with several promising agents emerging beyond OCA. Seladelpar and elafibranor have demonstrated favorable biochemical efficacy, safety, and tolerability, particularly in terms of pruritus improvement, and have recently gained FDA approval. Fenofibrate, while not officially approved for PBC, remains a practical and accessible adjunct in patients with incomplete UDCA response. However, robust long-term data on clinical outcomes such as liver transplantation or survival remains lacking for all agents. Given the heterogeneous response and safety concerns, especially in patients with cirrhosis, treatment selection must be tailored, incorporating disease stage, comorbidities, and patient preferences. Future research should prioritize head-to-head comparisons and long-term outcome data to better inform evidence-based decision-making in PBC management. Until robust transplant-free survival data emerges, physicians must integrate surrogate responses, symptom relief, safety signals, and risk prediction. Regular therapy reassessment and participation in trials or registries remain essential to advance outcome-based care

## Figures and Tables

**Table 1:** Key Features of Obeticholic acid, Fibrates, Seladelpar, and Elafibranor in PBC Management.

Feature	Obeticholic Acid	Bezafibrate/Fenofibrate	Seladelpar	Elafibranor
DOSE [14,15,16,17]	5–10 mg daily	145–200 mg daily	10 mg daily	80 mg daily
ALP NORMALIZATION (%) [14,15,16,17]	~15	~50 reduction; 30–40 normalization in some studies	25–27.3	~15
EFFECT ON PRURITUS [14,15,16,17]	Worsens (dose-dependent)	Possible improvement (bezafibrate > fenofibrate)	Significant improvement	Neutral/mild
COMMON AES [14,15,16,17]	Pruritus, fatigue	GI upset, ↑ creatinine, myopathy	GI discomfort, headache	GI symptoms, myalgia
CONTRAINDICATIONS [18,19,20,21]	Decompensated cirrhosis, portal hypertension	Severe renal/liver disease, gallbladder disease	Decompensated cirrhosis, biliary obstruction	Pregnancy, biliary obstruction
REGULATORY STATUS [5,22,23,24]	FDA-approved (2016)	Off-label in US	FDA-accelerated approval (2024)	FDA-accelerated approval (2024)

AASLD: American Association for the Study of Liver Diseases, AES: adverse events, ALP: alkaline phosphatase, FDA: U.S. Food and Drug Administration, FXR: farnesoid X receptor, GI: gastrointestinal, NRS: numerical rating scale, PPAR: peroxisome proliferator-activated receptor, OCA: obeticholic acid, PBC: primary biliary cholangitis, UDCA: ursodeoxycholic acid.
